# Multifaceted Nanocomposites Combining Phosphorylated PVA, MXene, and Cholesteric Liquid Crystal: Design and Application Insights

**DOI:** 10.3390/nano15161251

**Published:** 2025-08-14

**Authors:** Tăchiță Vlad-Bubulac, Diana Serbezeanu, Elena Perju, Dana Mihaela Suflet, Daniela Rusu, Gabriela Lisa, Tudor-Alexandru Filip, Marius-Andrei Olariu

**Affiliations:** 1 Petru Poni Institute of Macromolecular Chemistry, Aleea Gr. Ghica Voda 41A, 700487 Iasi, Romania; diana.serbezeanu@icmpp.ro (D.S.); elena.perju@icmpp.ro (E.P.); dsuflet@icmpp.ro (D.M.S.); rusu.daniela@icmpp.ro (D.R.); 2 Department of Chemical Engineering, Faculty of Chemical Engineering and Environmental Protection, Gheorghe Asachi Technical University of Iasi, Bld. Prof. Dr. Doc. D. Mangeron 73, 700050 Iasi, Romania; gapreot@yahoo.com; 3 Department of Electrical Measurements and Materials, Gheorghe Asachi Technical University of Iasi, Bld. Prof. Dr. Doc. D. Mangeron 67, 700050 Iasi, Romania; tudor-alexandru.filip@academic.tuiasi.ro (T.-A.F.); molariu@tuiasi.ro (M.-A.O.)

**Keywords:** phosphorylated PVA, MXene, cholesteric liquid crystal, multifunctional nanocomposites

## Abstract

In this study, composite films based on phosphorylated polyvinyl alcohol (PVA-P), Ti_3_C_2_T_x_ MXene, and cholesteryl acetate (ChLC) were designed and characterized to explore their potential in flexible electronic applications. The incorporation of phosphate groups and ChLC enhanced intermolecular interactions, as confirmed with FTIR spectroscopy. Morphological and optical analyses revealed a transition from homogeneous to phase-separated structures with birefringent textures in ChLC-rich films. Thermal studies demonstrated improved stability and increased glass transition and melting temperatures, particularly in samples with higher ChLC content. Mechanical and dielectric evaluations highlighted the tunability of stiffness, flexibility, permittivity, and dielectric losses depending on MXene and ChLC ratios. These multifunctional films exhibit flame-retardant behavior and show promise for use in stimuli-responsive, sustainable electronic devices such as flexible displays and sensors.

## 1. Introduction

Progress and innovation in materials science increasingly rely on the development of next-generation materials that integrate multifunctionality, sustainability, and responsiveness to external stimuli such as heat, light, or mechanical stress [1,2,3,4]. These features are essential to meet the escalating demands for high-performance materials in advanced applications including flexible electronics [5,6], advanced sensing technologies [7,8], and smart integrated platforms [9,10]. This quest compels the engineering of materials that not only exhibit synergistic assemblage of thermal, mechanical, electrical, and optical properties but also support the Green Deal paradigms, by mitigating ecological footprints through biodegradable, recyclable, or renewable compositions [11,12]. To achieve such objectives, interdisciplinary approaches, forefront synthesis techniques, and nanoscale engineering are required to tailor material architectures at the molecular and atomic levels, thereby optimizing performance while aligning with the principles of circular economy [13].

Polyvinyl alcohol (PVA), a synthetic polymer renowned for its multifaceted versatility, comes forth as an exemplary polymer matrix for such onward progress due to its exceptional mechanical robustness, inherent biodegradability, and remarkable chemical tunability [14]. Synthesized through the controlled hydrolysis of polyvinyl acetate, PVA possesses a linear macromolecular backbone abounding in hydroxyl groups, which facilitates further chemical functionalization and fosters extensive intermolecular hydrogen-bonding networks [15,16]. These attributes underpin the capacity of PVA to form transparent, mechanically stable, and homogeneous films. Furthermore, PVA’s compatibility with an extended spectrum of additives, such as metallic nanoparticles [17,18], carbon-based nanostructures [19], and two-dimensional (2D) nanomaterials like graphene [20] or transition metal carbides (MXene) [21,22], recommends it as a platform of choice for engineering multifunctional nanocomposites [23]. However, pristine PVA lacks intrinsic flame resistance and high functional integration, which limits its application in high-performance electronics. To address these limitations, phosphorylation of PVA introduces phosphorus-containing groups that enhance thermal stability, flame retardancy, and mechanical integrity through stronger intermolecular interactions and crosslinking [24,25,26,27,28]. Nevertheless, phosphorylated PVA alone may not provide the necessary electrical functionality or structural reinforcement required for multifunctional devices.

The incorporation of Ti_3_C_2_T_x_ MXene, a 2D transition metal carbide with outstanding electrical conductivity and mechanical strength, offers a strategic pathway to enhance the functional capabilities of PVA-based systems [15,29,30]. The surface terminations of MXenes (–OH, –O–, –F) promote strong interfacial bonding with phosphorylated PVA, improving dispersion and structural coherence. Additionally, recent studies have highlighted MXene’s ability to form ordered structures in dispersions, driven by its anisotropic two-dimensional (2D) morphology, surface chemistry, and inter-flake interactions [31,32,33]. This self-organization behavior, often observed in aqueous or polar solvent dispersions, results in liquid crystalline phases or aligned assemblies, which significantly influence the microstructural organization and properties of MXene-based composites [34,35,36].

Cholesteric liquid crystals (ChLCs) are a class of soft matter characterized by their distinctive helical molecular organization, which imparts unique optical and physical properties, making them a subject of increasing scientific interest across diverse fields [37,38]. Polymer-dispersed cholesteric liquid crystals (PDChLCs) constitute a sophisticated subset of liquid crystal (LC)/polymer composites, distinguished by their microphase-separated morphology, where ChLC droplets are homogeneously encapsulated within a porous polymer matrix [39,40]. Thanks to their facile preparation, excellent film-forming ability, and stable optical characteristics, polymer-dispersed liquid crystals (PDLCs) are widely employed in diverse applications, including switchable windows for energy-efficient buildings [41], reflective displays for low-power electronic devices, tunable microlenses, and optical shutters for privacy or light filtering [40].

Despite significant progress in developing individual PVA-based composites, MXene-polymer hybrids, or liquid crystal–polymer systems, the synergistic integration of phosphorylated PVA, Ti_3_C_2_Tx MXene, and ChLCs remains largely unexplored. The research gap lies in understanding how these three distinct components interact at the molecular, interfacial, and macroscopic levels to generate materials with emergent, multifunctional behavior.

In this work, we propose a novel triadic hybrid system comprising phosphorylated PVA as the functional matrix, Ti_3_C_2_Tx MXene as the conductive and reinforcing phase, and cholesteryl acetate as the optically active liquid crystal. This unique combination aims to overcome current limitations in polymer nanocomposites by enabling tunable optical, dielectric, mechanical, and thermal properties within a sustainable platform. We investigate the structural organization, interfacial interactions, and resulting multifunctionality of these composites, with a view toward next-generation applications such as stimuli-responsive electronics, flexible sensors, and smart visual interfaces.

## 2. Materials and Methods

### 2.1. Materials

PVA powder (Mw = 30,000–70,000 Da, degree of hydrolysis = 87–90%) from Sigma-Aldrich Chemie GmbH (Taufkirchen, Germany) was utilized to prepare PVA-P via the controlled nucleophilic substitution reaction, wherein the hydroxyl (-OH) groups on the PVA backbone were reacted with the chlorine (-Cl) atoms of phenyl dichlorophosphate, following a protocol established in our previous publications [24,42]. Phenyl dichlorophosphate (purity = 98%) was sourced from TCI Europe (Zwijndrecht, Belgium). *N, N-*Dimethylformamide (DMF) was obtained from Sigma-Aldrich (Taufkirchen, Germany). Cholesteryl acetate and LiCl were purchased from Sigma-Aldrich (Steinheim, Germany). Ti_3_AlC_2_ MAX phase powder with a particle size < 40 µm has been acquired from carbon (Ukraine). The minimally intensive layer delamination method MILD (via LiF-HCl etching) with in situ generation of HF, was applied to obtain Ti_3_C_2_T_x_ multilayered MXene, while the delamination procedure used Li^+^ as intercalant [43]. The preparation and characterization of Ti_3_C_2_T_x_ MXene were described in our previous papers [34,35].

### 2.2. Fabrication of PVA-P Film (P0), PVA-P/MXene (P1, P2), and PVA-P/MXene/ChLC Composites (P3–P5)

**Scheme 1:**
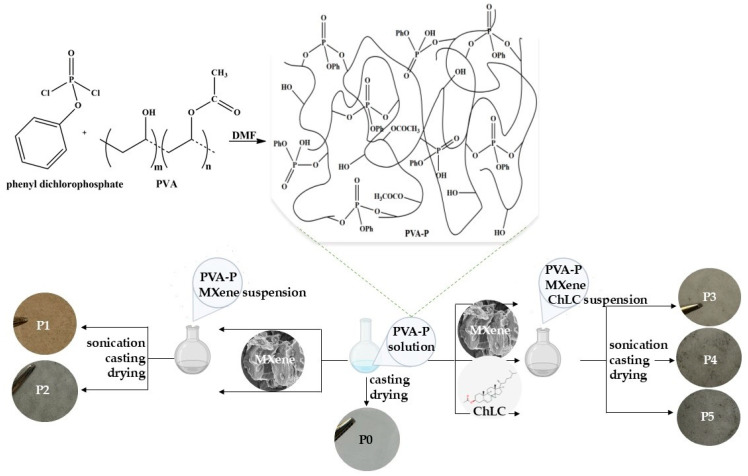
Schematic representation regarding the preparation of PVA-P-based nanocomposites.

The resulting PVA-P, likely featuring phosphodiester linkages between the PVA backbone and phenyl phosphate groups (Scheme 1), introduces enhanced functionalities, including flame retardancy and thermal stability, critical for advanced applications. Post-reaction purification of PVA-P was performed through dialysis against distilled water over five days to remove unreacted phenyl dichlorophosphate, DMF, and low-molecular-weight byproducts. Then, the product was dried at 60 °C under vacuum in an oven.

The PVA-P stock solution was prepared by dissolving phosphorylated PVA powder (PVA-P) at a 10% *w*/*v* concentration in double-distilled water, followed by continuous magnetic stirring at room temperature for 24 h to ensure complete dissolution and a homogeneous solution. The blank sample, denoted P0, was fabricated by casting a specific volume of the PVA-P stock solution onto a Petri dish and then drying it under ambient conditions (room temperature, ~25 °C) for 72 h, yielding a transparent, mechanically stable film (Scheme 1). For composite samples P1 to P5, varying amounts of Ti_3_C_2_T_x_ MXene were incorporated into the PVA-P solution, according to Table 1.

The MXene was uniformly dispersed through magnetic stirring for 30 min, promoting strong interfacial interactions with PVA-P’s phosphate groups, which facilitate stable nanofiller dispersion. For samples P3 to P5, a cholesteryl acetate (ChLC) solution (1% *w*/*v*) in ethanol, a cholesteric liquid crystal derivative, was additionally introduced, and the mixtures underwent further stirring for 30 min to ensure homogeneity, followed by a 2-h degassing step using an ultrasonic bath at room temperature to eliminate entrapped air bubbles, which could otherwise compromise film uniformity and optical clarity (Scheme 1, Table 1). The degassed solutions were then cast onto Petri dishes in controlled volumes to form thin films, dried under ambient conditions for 72 h to achieve stable, defect-free films suitable for further characterization.

### 2.3. Methods

#### 2.3.1. FTIR Investigation

The chemical structure and molecular interactions of the investigated phosphorylated polyvinyl alcohol (PVA-P)-based composite films, incorporating Ti_3_C_2_T_x_ MXenes and cholesteryl acetate (ChLC), were examined using Fourier Transform Infrared (FTIR) spectroscopy performed on a LUMOS Microscope FTIR spectrophotometer (Bruker Optik GmbH, Ettlingen, Germany), equipped with an attenuated total reflection (ATR) accessory, which enables direct, non-destructive examination of solid films by minimizing sample preparation. Spectra were acquired over a broad wavenumber range of 4000–500 cm^−1^, encompassing key vibrational modes such as O–H, C–H, P–O, and C=O stretches, with a spectral resolution of 4 cm^−1^ to achieve precise peak differentiation.

#### 2.3.2. Morphology of PVA-P Film, PVA-P/MXene Films, and PVA-P/MXene/ChLC Composites

The surface morphology and microstructural characteristics of the phosphorylated polyvinyl alcohol (PVA-P)-based composite films, incorporating Ti_3_C_2_T_x_ MXenes and cholesteryl acetate (ChLC) (samples P1–P5), were analyzed using a Verios G4 UC Scanning Electron Microscope (SEM) (Thermo Scientific, Czech Republic), a high-resolution instrument optimized for nanoscale imaging. To mitigate charge accumulation and enhance electrical conductivity during electron beam exposure, the films were coated with a 6 nm layer of platinum using a Leica EM ACE200 Sputter Coater (Leica Mikrosysteme GmbH, Vienna, Austria). This ultrathin platinum coating ensures minimal interference with surface topography while providing sufficient conductivity to prevent imaging artifacts, enabling clear visualization of the composite’s microstructural features. The SEM analysis was performed in high vacuum mode, which eliminates atmospheric interference and enhances resolution, utilizing a secondary electron detector (Everhart-Thornley detector, ETD) to capture topographical details with high contrast. An accelerating voltage of 5 kV was employed, balancing the need for sufficient electron penetration to reveal surface and near-surface features with the prevention of beam-induced damage to the polymer matrix.

#### 2.3.3. Optical Microscopy (POM)

The surface appearance and optical characteristics of the samples P0–P5 were investigated with polarized optical microscopy, under perpendicular cross-polarizers, using a Zeiss Microscope Axio Imager A2M (Carl Zeiss Microscopy GmbH, Oberkochen, Germany). This microscope was equipped with a Linkam Plate LTS420 (Honeycrock Lane, Salfords, UK), a precision temperature-controlled stage capable of maintaining stable thermal conditions (ranging from ambient temperature to 120 °C), which facilitated in situ observation of the films’ optical behavior under varying temperatures, particularly relevant for assessing ChLC’s thermotropic properties. The use of a 10× objective and a 10 °C/min heating-cooling rate enabled multi-scale imaging, capturing both macroscopic film uniformity and microscopic features, such as MXene dispersion, ChLC’s chiral organization, and the PVA-P matrix’s microstructure. Observations were conducted in polarized light modes, leveraging the Axio Imager A2M’s polarizing capabilities to highlight the birefringence of ChLC’s helical structure.

#### 2.3.4. Thermogravimetric Analysis (TGA)

The thermal stability and decomposition behavior of the samples P0–P5 were rigorously evaluated using a Mettler Toledo TGA-SDTA851e thermogravimetric analyzer (Columbus, OH, USA), the experiments being conducted under a nitrogen atmosphere to prevent oxidative degradation. A gas flow rate of 20 mL·min^−1^ was maintained to provide a consistent inert environment, minimizing residual oxygen and facilitating the removal of volatile decomposition products. The measurements were performed under dynamic conditions with a heating rate of 10 °C/min, over a temperature range of 25–650 °C. Sample masses ranged from 2.34 mg to 3.89 mg.

#### 2.3.5. Differential Scanning Calorimetry (DSC)

The thermal transitions of the films were investigated using a Mettler Toledo DSC 1 calorimeter (Mettler Toledo, Greifensee, Switzerland). The experiments were conducted under a nitrogen atmosphere with a flow rate of 150 mL·min^−1^, ensuring an inert environment to prevent oxidative degradation. A heating rate of 10 °C·min^−1^ was applied, consistent with the thermogravimetric analysis (TGA) protocol previously described. The temperature range was from ambient conditions to 150 °C, a chosen range for polymer composites to capture key transitions such as *T_g_*, *T_m_*, and phase changes associated with chosen ingredients, without exceeding much the onset of significant decomposition (~130 °C for PVA-P). Samples of masses ranging from 2.35 to 3.17 mg were prepared and placed in aluminum crucibles with perforated lids, a configuration designed to allow the release of volatile compounds (e.g., residual water or decomposition byproducts) while maintaining thermal contact with the calorimeter’s sensors.

#### 2.3.6. Mechanical Properties

The mechanical properties of the samples were systematically evaluated at room temperature using a Brookfield Texture PRO CT3^®^ Texture Analyzer (Brookfield Engineering Laboratories Inc., Middleborough, MA, USA). The testing adhered to the ASTM D882-10 Standard Test Method [44] for tensile properties of thin plastics, ensuring standardized and reproducible results. Two distinct mechanical tests—uniaxial tensile testing and penetration testing—were conducted to assess the films’ strength, stiffness, toughness, and resistance to puncture. Uniaxial tensile tests were performed using the TA-RCA module, specifically designed for thin films, on rectangular samples with dimensions of 40 mm length, 10 mm width, and ~0.10 mm thickness, reflecting the thin, flexible nature of the PVA-P-based films. A trigger load of 0.067 N was applied to initiate the test, ensuring accurate detection of the onset of deformation, while a tensile rate of 0.5 mm/s was maintained to provide controlled elongation, allowing precise measurement of stress–strain behavior. The Young’s modulus (Y), a measure of the films’ stiffness, was calculated from the slope of the stress–strain curves within the linear elastic region, specifically between 0.5% and 4% elongation, a range chosen to capture the initial elastic response while avoiding artifacts from sample alignment or slippage. The tests continued until specimen fracture, providing data on ultimate tensile strength (UTS), elongation at break, and other parameters. Penetration tests were conducted on rectangular samples measuring 15 mm × 15 mm, using a TA-FSF probe with a blunt head to simulate localized puncture resistance, a critical property for flexible electronic substrates exposed to mechanical stress. A penetration force of 44 N was applied at a test speed of 4 mm/s, allowing controlled deformation and accurate measurement of the force required to puncture the films. The tests terminated upon specimen fracture, providing insights into the films’ toughness and resistance to mechanical failure. The penetration test results complement tensile data, highlighting the composites’ suitability for applications requiring durability under localized stress, such as wearable electronics or protective coatings.

#### 2.3.7. Electrical Properties

The dielectric properties of the phosphorylated polyvinyl alcohol (PVA-P)-based composite films, incorporating Ti_3_C_2_T_x_ MXenes and cholesteryl acetate (ChLC), were further characterized using a broadband dielectric spectrometer (Novocontrol Technologies, Montabaur, Germany), an instrument designed for high-precision measurement of dielectric permittivity, conductivity, and relaxation processes across a wide frequency range.

## 3. Results

### 3.1. FTIR Analysis

**Figure 1:**
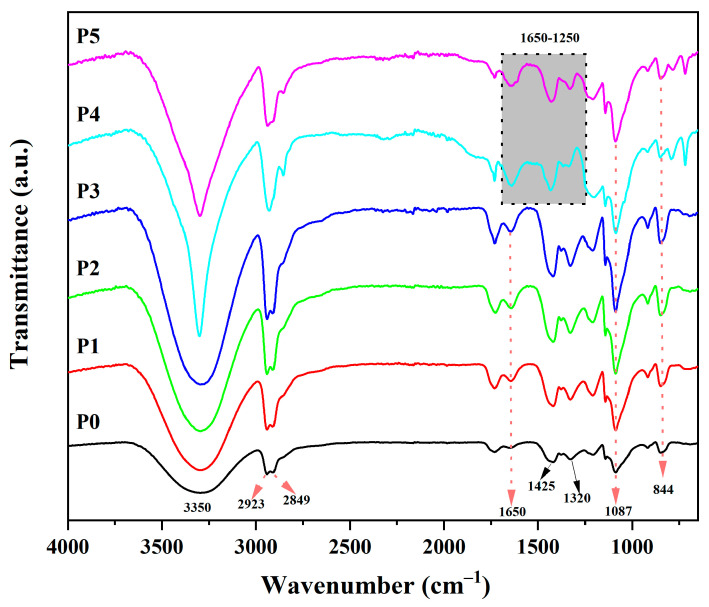
FTIR spectra of the investigated composite films.

The formation of the P0 network was confirmed with FTIR spectroscopy, as shown in Figure 1. The FTIR spectrum of P0 exhibits a broad and intense absorption band centered at 3350 cm^−1^, corresponding to the stretching vibrations of non-condensed hydroxyl groups still present along the macromolecular chain. Characteristic absorption bands of aliphatic C–H bonds are observed at 2923 and 2849 cm^−1^ (asymmetric and symmetric stretching vibrations), and at 1425 cm^−1^ (bending vibrations). A distinct band at 1728 cm^−1^ is attributed to residual acetate groups [45]. In addition, absorption bands around 1256 cm^−1^ are attributed to alkyl phosphates ((RO)_3_P=O), while weaker bands near 1320 cm^−1^ correspond to aryl phosphate groups ((ArO)_3_P=O). Bands observed at approximately 1650 cm^−1^ suggest the presence of acid phosphate structures, such as (RO)_2_(HO)P=O and/or (ArO)_2_(HO)P=O.

Furthermore, bands at 1087 cm^−1^ and 844 cm^−1^, associated with asymmetric and symmetric stretching of P–O–C linkages, respectively, confirm the formation of phosphate ester bonds. These spectral features collectively support the successful chemical crosslinking of PVA chains via phenyl dichlorophosphate condensation. Given its bifunctional nature, phenyl dichlorophosphate effectively facilitates network formation by introducing a variety of aryl and acid phosphate linkages, as illustrated schematically in Scheme 1. With the incorporation of MXene (samples P1 and P2), subtle shifts and increased definition in the O–H and phosphate-related regions suggest enhanced interaction between MXene and the PVA-P matrix, likely via hydrogen bonding and possible coordination with phosphate groups. Nonetheless, due to the presence of MXenes in the system—compounds rich in –OH, –F, and –O surface groups formed during exfoliation and intercalation—the FTIR spectra of P1 and P2 exhibit broadened O–H stretching bands, slightly shifted toward 3400 cm^−1^ (Figure S1, Supplementary Information). Additionally, the characteristic band at 1087 cm^−1^ becomes more intense, attributed to Ti–O or Ti–O–Ti stretching vibrations. A subtle signal around 619 cm^−1^ confirms the presence of Ti–F groups, further supporting the successful integration of MXene particles into the polymer matrix. Samples P3–P5, which contain both MXene and the liquid crystal additive ChLC, display further spectral changes. Thus, overlapped characteristic bands for Ti_3_C_2_T_x_ MXene appeared at ~3400 cm^−1^ (contribution of O–H from surface, Figure S1 Supplementary Information) along with the influences of O–H and C–H interactions coming from the polar, chiral structure of ChLC, which contains acetate and cholesterol side chains, these contributions being more relevant in the samples P4 and P5 when the content of ChLC increased. MXene’s characteristic Ti–O band at ~1100 cm^−1^ shifts slightly in P3–P5, suggesting coordination or interaction with the phosphate groups of the PVA-P matrix ((RO)_3_P=O at 1256 cm^−1^). The presence of ChLC appears to enhance these interactions, as evidenced by variations in the 1650–1250 cm^−1^ region, where additional contributions from ChLC’s functional groups (imine or aromatic phosphate moieties) are observed. These changes indicate that ChLC may act as a compatibilizer, facilitating stronger interfacial bonding between MXene and the phosphate groups of PVA-P.

### 3.2. Morphological Characteristics

**Figure 2:**
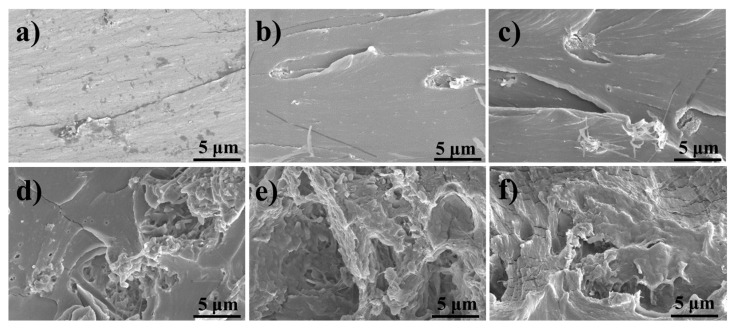
SEM images of the investigated composite films P0 (**a**), P1 (**b**), P2 (**c**), P3 (**d**), P4 (**e**), P5 (**f**).

Figure 2 displays SEM images of the cross-sectional morphology of composite films (P0–P5). At no MXene content and low MXene content (Figure 2a,b), the films exhibit smooth and uniform surfaces, indicating that the PVA-P matrix forms a continuous phase with well-dispersed MXene sheets. The absence of visible phase boundaries or agglomerates suggests strong compatibility and efficient dispersion at these concentrations.

**Figure 3:**
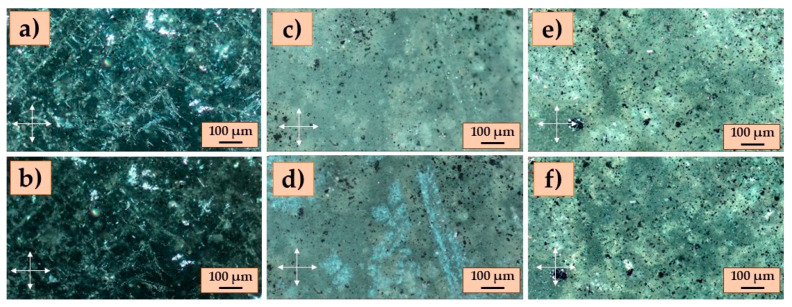
POM microphotographs taken for the samples containing ChLC during the first heating cycle: (**a**) P3 at 54.3 °C; (**b**) P3 at 120.1 °C; (**c**) P4 at 26.6 °C; (**d**) P4 at 107 °C; (**e**) P5 at 38.2 °C; and (**f**) P5 at 120.1 °C.

As the MXene content increases (Figure 3c), layered and overlapping sheet structures become more apparent, reflecting the intrinsic lamellar morphology of MXene. While dispersion remains relatively uniform, the emergence of microvoids and localized accumulations indicates the initial stages of agglomeration at higher MXene loadings. With the incorporation of ChLC into the PVA-P/MXene composites (Figure 2d–f), the morphology changes markedly. The cross-sections display increased surface roughness, numerous voids, microcracks, and evidence of phase heterogeneity. These features suggest poor interfacial adhesion between ChLC and the PVA-P/MXene matrix, due to its scarce solubility in the host fluid, resulting in brittle fracture behavior and structural discontinuities. The observed morphological defects likely arise from phase separation and inadequate interaction at the polymer–ChLC interface, further compromising the mechanical integrity of the composite films. SEM images (Figure 2d–f) reveal that ChLC in P3–P5 is dispersed as phase-separated microdomains, with increased surface roughness, voids, and microcracks indicating poor interfacial adhesion due to ChLC’s limited solubility in the polar PVA-P/MXene matrix.

### 3.3. Optical Microscopy Observations

POM introspection of the blank sample, denoted P0, consisting solely of phosphorylated polyvinyl alcohol (PVA-P) without the incorporation of Ti_3_C_2_T_x_ MXenes or cholesteryl acetate (ChLC), exhibited expected transparency, under non-polarized mode, P0 appearing as a colorless, smooth, and uniform film with minimal light scattering, indicating a smooth, defect-free surface (Scheme 1, the inset for P0). However, under polarized light, sample P0 revealed a morphology characterized by acicular (needle-like) formations (Figure S2, Supplementary Information), suggesting the presence of internal ordering or oriented crystalline domains within the phosphorylated matrix. These effects can induce localized crystalline domains or oriented structures. Under polarized light, such domains may appear as acicular (needle-like) formations, which are not visible under non-polarized light due to the otherwise uniform and transparent nature of the film [46].

The microscopy observations upon samples having no content of ChLC (P1, P2) revealed surface morphologies with reduced transparency and increased light scattering due to MXene’s dark, opaque appearance and nanoscale aggregation, observable along the whole microscopic view, as homogeneously dispersed granular formations, especially at higher loadings (Figures S3 and S4, Supplementary Information).

Figure 3 illustrates polarized optical microscopy images depicting the texture morphology of composite films P3, P4, and P5, which incorporate varying concentrations of cholesteryl acetate (ChLC), in conjunction with Ti_3_C_2_T_x_ MXenes within the phosphorylated polyvinyl alcohol matrix. The POM analysis revealed distinct temperature-dependent microstructural dynamics in these ChLC-containing composites. Above the glass transition temperature (*T_g_*, 68–74 °C for P3–P5), heating induced subtle softening or enhanced polymer chain mobility, manifested as refined textural changes under polarized light, likely due to increased segmental flexibility within the PVA-P matrix, as corroborated by differential scanning calorimetry data.

The POM images of P3–P5 (Figure 3) show birefringent textures due to ChLC’s chiral molecular structure, but lack characteristic cholesteric LC textures (fingerprint or focal conic patterns) [47,48]. This is attributed to ChLC’s confinement within the rigid PVA-P/MXene matrix, which disrupts long-range helical ordering. Phase separation (SEM: voids, microcracks) and steric constraints from the phosphorylated network (FTIR: phosphate bands) limit ChLC to localized birefringent microdomains. The absence of a cholesteric-to-isotropic transition within the studied temperature range (DSC: *T_m_* = 112 °C for P4) further stabilizes these domains, preserving birefringence without forming macroscopic cholesteric textures. The birefringent patterns exhibited amplified molecular mobility above 100 °C, reflecting the dynamic reorientation of ChLC’s helical domains under thermal stimulation. However, within the investigated temperature range, higher than the ChLC isotropic transition, no phase transitions accompanied by perceptible color shifts or significant alterations in birefringence were observed, indicating that the cholesteric-to-isotropic phase transition of ChLC was not attained [49]. POM birefringent textures (Figure 3) confirm ChLC’s anisotropic domains, but their non-uniformity suggests confined microdomains rather than a continuous liquid crystalline phase, with domain size increasing from P3 to P5 due to higher ChLC content. This thermal stability of the optical textures highlights the effective stabilization of ChLC within the PVA-P/MXene matrix, preserving its thermotropic optical functionality and reinforcing the composites’ suitability for advanced electronic applications, such as stimuli-responsive displays, colorimetric sensors, or flexible photonic devices, where consistent birefringence under moderate thermal conditions is essential [50].

### 3.4. Thermal Stability and Decomposition Behavior

**Figure 4:**
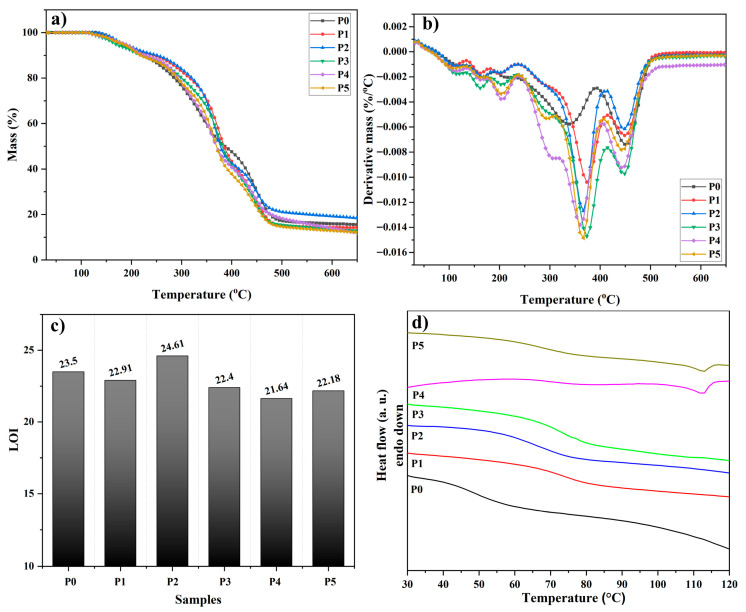
Thermogravimetric analysis (TG) (**a**) and derivative thermogravimetric (DTG) curves (**b**), calculated Limiting Oxygen Index (LOI) values derived from char yields at 650 °C (**c**), and Differential Scanning Calorimetry (DSC) curves of the samples of the investigated composite films (P0–P5) (**d**).

Thermogravimetric analysis (TGA) and derivative thermogravimetry (DTG) were performed to investigate the thermal stability and decomposition mechanisms of the composite films (P0–P5). The thermal degradation profiles of the composite films are shown in Figure 4a,b, while detailed thermal parameters are summarized in Table 2. The TGA curves of samples P0 to P2 exhibited five distinct mass loss events, whereas P3 to P5 demonstrated six, reflecting the addition of ChLC and its contribution to thermal behavior. The first mass loss, observed around 105 °C for all samples, is attributed to the evaporation of physically adsorbed moisture. The second stage, occurring between 157–167 °C, corresponds to the removal of bound or intrinsic water, often retained within the polymer network or between layered components. The third stage, detected in the range of 201–210 °C, is likely associated with the initial thermal degradation of the polyvinyl alcohol (PVA) matrix, involving chain scission and elimination reactions leading to the formation of volatile products such as water, acetic acid, or other small molecules. This is consistent with earlier studies on partially hydrolyzed PVA [24,51]. In samples P3 to P5, which contain ChLC, an additional degradation event is observed in the range of 286–304 °C. This stage is attributed to the thermal decomposition of ChLC, involving the breakdown of its cholesteric units. In particular, the presence of aromatic or rigid mesogenic units (from ChLC) could locally increase the order of the system, contributing to nucleation phenomena or char formation during decomposition. However, the degradation stage observed between 286–304 °C for samples containing ChLC (P3–P5) is attributed to the thermal breakdown of cholesteryl acetate, which does not significantly enhance thermal stability but rather introduces an additional thermal event. The presence of this stage confirms the successful incorporation of ChLC into the composite matrix. The main decomposition step, between 336–373 °C, corresponds to the concurrent degradation of MXene and further breakdown of the PVA matrix, likely involving dehydroxylation processes and the decomposition of the MXene surface functional groups (e.g., –OH, –F, –O). A second major degradation stage, occurring between 444–450 °C, is attributed to the final decomposition of the polymer backbone, representing carbonization and chain scission of the remaining organic material. The main contributors to enhanced thermal stability appear to be the inorganic MXene sheets, whose barrier effect is reflected in the increased residual mass at 650 °C (Supplementary Note S1). The char yield values of up to 17.77% in samples with higher MXene content (e.g., P2 and P5) confirm this effect. This suggests that the thermal behavior is governed primarily by the filler content and its interaction with the polymer matrix rather than by extensive chemical crosslinking.

The materials under investigation exhibited high Limiting Oxygen Index (LOI) values, which are key indicators of flame retardancy. The LOI values were estimated using the linear equation LOI = 17.5 + 0.4 × CY, where CY represents the char yield at 650 °C. Based on this relationship, the LOI values of the samples ranged from 22.18 to 24.61, with sample P2 showing the highest value (Figure 4c). The combination of these compounds significantly influences their thermal and flame-retardant properties. In particular, the incorporation of MXene into the PVA-P matrix enhances both thermal stability and flame retardancy [52].

### 3.5. DSC Analysis

Differential scanning calorimetry (DSC) was employed to assess the thermal transitions of the phosphorylated polyvinyl alcohol (PVA-P)-based composite films (P0–P5), focusing on the glass transition temperature (*T_g_*) and melting temperature (*T_m_*), in accordance with light of polarized optical microscopy (POM) observations for samples P3–P5 containing cholesteryl acetate (ChLC) (Figure 4d). The DSC analysis revealed *T_g_* values ranging from 51 °C for the blank PVA-P film (P0) to 74 °C for samples P1 and P3, which incorporate Ti_3_C_2_T_x_ MXenes and, in P3, ChLC. In P1, *T_g_* increases from 51 °C (P0) to 74 °C due to MXene’s 2D structure and strong interactions with PVA-P phosphate groups (FTIR: ~3400 cm^−1^ O–H, ~1100 cm^−1^ Ti–O, ~1256 cm^−1^ P=O), restricting chain mobility. Despite ChLC’s plasticizing potential, its low content in P3 forms ordered microdomains (POM: birefringent textures) stabilized by the MXene–PVA-P network (FTIR: 1087, 844 cm^−1^ P–O–C). This balance offsets plasticization, keeping the glass transition at 74 °C, with MXene’s stiffening effect (E = 5.88 MPa in P3) counteracting ChLC’s mobility enhancement [29]. Further increasing the content of ChLC in P4 and 5, *T_g_* drops to 70 and 68 °C, respectively, due to ChLC’s plasticizing effect, reduced crosslinking density, and possible phase separation, which together enhance polymer chain mobility and decrease thermal rigidity. The POM images of P3–P5, showing birefringent textures indicative of ChLC’s chiral helical structure, support these interactions, as the microphase-separated domains observed under polarized light, up to 120 °C suggest ordered ChLC integration that further constrains polymer chain dynamics, particularly above 100 °C where molecular mobility intensifies without altering birefringence [53,54]. Melting behavior was exclusively observed in samples P4 and P5, with *T_m_* values of 112 °C and 113 °C, respectively, suggesting partial crystallinity induced by higher ChLC content, likely due to phase separation of ChLC’s helical domains, as evidenced by the stable birefringent textures in POM images that persist without color shifts or cholesteric-to-isotropic transitions within the tested range.

### 3.6. Mechanical Analysis

**Figure 5:**
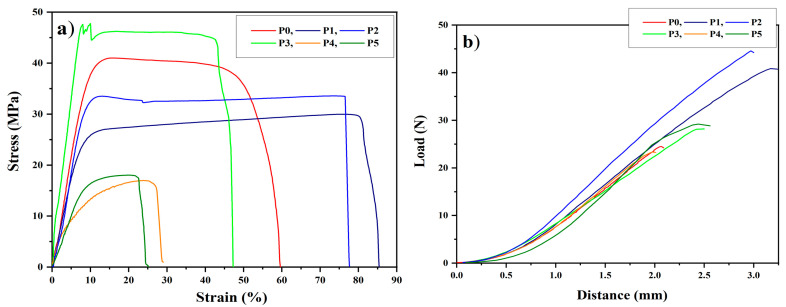
(**a**) The stress–strain curves and (**b**) load force as a function of distance curves for the studied composites.

The mechanical properties of the composites in the series were performed, the mechanical behavior of the composites is shown in Figure 5, while the data are presented in Table 3 which provides insights into Young’s modulus (Y), ultimate tensile strength (σ_ult_), fracture strain (ε_f_), yield strain (ε_y_), yield stress (σ_0.2_%), modulus of resilience (M_R_), modulus of toughness (M_T_), absorbed energy until fracture, and penetration force. These properties reflect the interplay of PVA-P’s phosphoester groups, MXene’s reinforcement, and ChLC’s chiral organization, as corroborated by FTIR, SEM, POM, TGA, and DSC analyses, informing their suitability for advanced applications.

The blank PVA-P sample with a Young’s modulus of 4.85 MPa, σ_ult_ of 40.18 MPa, and ε_f_ of 49.00%, exhibits moderate stiffness and strength, driven by phosphoester-induced hydrogen-bonding, but lower stiffness than pristine PVA (~500 MPa) due to plasticization. Its high M_R_ (168.25 kJ/m^3^) and M_T_ (1417.92 kJ/m^3^) indicate good resilience and toughness, suitable for flexible substrates, while a penetration force of 24.48 N reflects moderate puncture resistance.

By combining MXene/PVA-P mass ratios of 20 mg/g and 40 mg/g, respectively, in samples P1 and P2, the obtained results of P1 (Y = 3.57 MPa, σ_ult_ = 29.96 MPa) and P2 (Y = 3.85 MPa, σ_ult_ = 33.47 MPa) showed reduced stiffness and strength compared to P0, despite MXene’s high intrinsic modulus (~0.484 TPa), likely due to microagglomeration disrupting the PVA-P matrix. Higher ε_f_ (80.04% for P1, 76.48% for P2) and M_T_ (2021.69–2252.19 kJ/m^3^) indicate enhanced ductility and toughness, driven by MXene’s layered structure. Penetration forces (40.86 N for P1, 44.59 N for P2) increase with MXene content, reflecting improved puncture resistance. These results are consistent with DSC’s *T_g_* increase to 74 °C, suggesting restricted chain mobility. The stress–strain curve of sample P3 (Figure 5a) shows an abnormal plateau region following the yield point, which is indicative of Lüders strain. This phenomenon, commonly associated with localized plastic deformation, may result from internal stress concentrations or heterogeneous filler distribution within the P3 composite. The observed behavior suggests that the P3 formulation facilitates strain localization before uniform plastic flow occurs, which differs from the deformation mechanisms seen in the other samples.

By integrating further the third ingredient, ChLC, into the composite P3 in a MXene/ChLC/PVA-P/mass ratio of 20 mg/20 mg/g, the mechanical properties resulted in the highest stiffness (Y = 5.88 MPa) and strength (σ_ult_ = 46.05 MPa), with ε_f_ of 42.10%, indicating a synergistic MXene-ChLC effect. POM’s birefringent textures confirm ChLC’s helical domains, enhancing matrix order, while DSC’s *T_g_* of 74 °C and FTIR’s C=O (~1730 cm^−1^) bands suggest strong interfacial interactions. High M_R_ (201.44 kJ/m^3^) and penetration force (29.81 N) support its robustness for sensors or EMI shielding.

Finally, continuing to increase the content of MXene and ChLC, in P4 and P5, resulted in the lowest stiffness and strength (P4 (Y = 1.63 MPa, σ_ult_ = 16.96 MPa) and P5 (Y = 2.05 MPa, σ_ult_ = 18.00 MPa)) with reduced ε_f_ (24.11% and 21.60%) and M_T_ (311.31–250.20 kJ/m^3^), due to ChLC’s plasticizing effect from phase-separated domains, as seen in POM and DSC’s *T_m_* (112–113 °C), indicating partial crystallinity. Penetration forces (23.31–29.20 N) remain comparable to P0, suggesting adequate puncture resistance for flexible displays. It is worth noting that while ChLC introduces chiral ordering that may act as a physical crosslinker at low concentrations (e.g., P3), higher loadings appear to plasticize the matrix and induce cavity formation, reducing mechanical strength. This dual behavior suggests that ChLC’s role is highly dependent on its concentration and interaction with both the polymer and MXene phases.

### 3.7. Electrical Analysis

**Figure 6:**
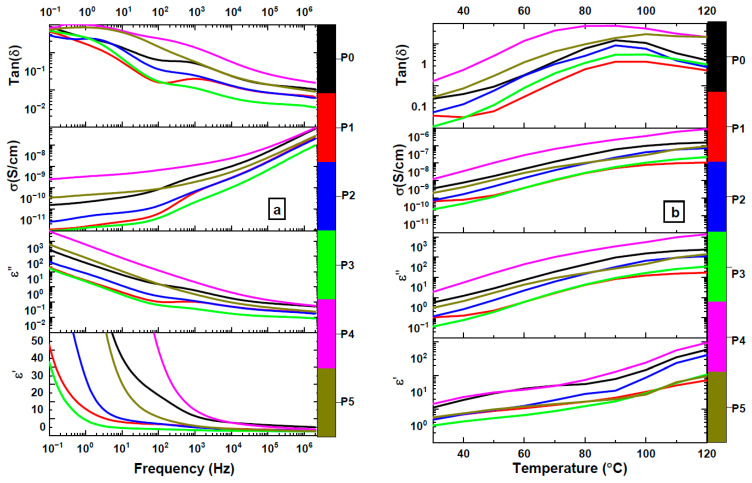
The dielectric permittivity (ε’), dielectric loss (ε”), conductivity (σ) and loss tangent (tan(δ)) of the composites as a function of frequency ranging from 10^−1^ Hz and 10^6^ Hz at room temperature (**a**) and function of temperature between 30 °C and 120 °C at 10^3^ Hz (**b**).

The dielectric characteristics of phosphorylated polyvinyl alcohol (PVA-P)-based composite films (P0–P5) containing Ti_3_C_2_T_x_ MXenes and cholesteryl acetate (ChLC) were assessed (Figure 6). Table 4 provides the real permittivity (ε’), imaginary permittivity (ε”), dielectric loss tangent (tan δ), and AC conductivity (σ) at 103 Hz. Due to polar phosphoester groups (FTIR: ~1250 cm^−1^ P=O), which contribute to dipole polarization, and relatively high losses (tan δ) from amorphous chain relaxations (DSC: Tg = 51 °C), P0 has moderate permittivity with ε’ = 11.1, ε” = 5.55, tan δ = 0.498, and σ = 5.50 × 10^−9^ S/cm. Because of its low conductivity, it can be used as a baseline dielectric matrix in an insulating polymer.

A consistent dielectric response is supported by the smooth, isotropic shape of POM. Despite MXene’s high conductivity (~7750 S/cm), P1 (ε’ = 5.2, ε” = 1.03, tan δ = 0.197, σ = 6.50 × 10^−10^ S/cm) and P2 (ε’ = 4.8, ε” = 1.14, tan δ = 0.236, σ = 7.23 × 10^−10^ S/cm) exhibit lower permittivity and losses when compared to P0. Reduced Young’s modulus (3.57–3.85 MPa) indicates low MXene loadings or agglomeration (SEM: granular patterns), which restricts the development of conductive networks. Higher MXene content is reflected in the modest σ rise in P2, although low values still show poor percolation. *T_g_* rising to 74 °C in DSC indicates little chain mobility, which lessens polarization. The lowest ε’ (3.3), ε” (0.35), tan δ (0.109), and σ (2.24 × 10^−10^ S/cm) are found in P3 (PVA-P/MXene/ChLC), suggesting a minimal dielectric response. This is probably because balanced MXene/ChLC interactions enhance matrix order (POM: birefringent textures) and restrict dipole mobility (DSC: Tg = 74 °C). It is appropriate for low-loss dielectric applications such as capacitors due to its high Young’s modulus (5.88 MPa), which indicates a stiff, ordered structure that reduces polarizability. P4 exhibits the highest ε’ (14.3), ε” (18.76), tan δ (1.307), and σ (1.18 × 10^−8^ S/cm). This is due to the high ChLC content that induces phase-separated domains (POM: stable birefringence, DSC: *T_m_* = 112 °C), which improves polarizability and conductive pathways. The elevated conductivity in P4 (σ = 1.18 × 10^−8^ S/cm) arises from the high ChLC content, which induces phase-separated domains (POM: stable birefringence, DSC: Tm = 112 °C). These domains enhance polarizability and create interfacial regions that facilitate charge transport via hopping or tunneling. Despite ChLC’s insulating nature, its chiral, polar structure amplifies the local electric field, synergizing with MXene’s conductive clusters to increase σ. This explains the higher dielectric response (ε’ = 14.3, ε” = 18.76) and losses (tan δ = 1.307) compared to P0–P3. The moderated values in P5 (ε’ = 5.7, ε” = 3.06, tan δ = 0.538, σ = 1.93 × 10^−9^ S/cm) point to an ideal ChLC/MXene ratio. Plasticization is indicated by low Young’s moduli (1.63–2.045 MPa), which correlate with higher dielectric losses and make them perfect for flexible, high-permittivity devices such as EMI shielding.

The low conductivity in P1 and P2 reflects poor MXene plate interconnection due to low loadings or agglomeration (SEM: granular patterns), hindering percolating networks. In P4, high ChLC content partially disperses MXene plates, forming localized conductive clusters that increase σ (1.18 × 10^−8^ S/cm). Microcavities, potentially arising from agglomeration (P1, P2) or phase-separated interfaces (P4), reduce effective permittivity and increase losses by introducing air gaps or charge-trapping sites. In P3, the ordered matrix (POM: birefringent textures, high Young’s modulus = 5.88 MPa) minimizes microcavities, ensuring low losses (tan δ = 0.109). These findings highlight the critical role of MXene dispersion and matrix morphology in optimizing dielectric performance.

These characteristics complement FTIR, SEM, POM, TGA, DSC, and mechanical investigations and indicate their suitability for electronic applications such as capacitive sensors or EMI shielding. They also show how the conductivity of MXene and the polar, chiral structure of ChLC affect the PVA-P matrix.

## 4. Discussion

This study demonstrates the successful development and comprehensive characterization of PVA-P-based nanocomposites incorporating MXene and cholesteryl acetate (ChLC), aimed at applications in advanced electronic devices. The integration of chemical, morphological, optical, thermal, mechanical, and dielectric analyses provides a holistic understanding of how MXene and ChLC modulate the matrix’s structure and performance.

- FTIR confirmed effective crosslinking in PVA-P and revealed hydrogen bonding and coordination between MXene surface groups and phosphoester moieties. ChLC introduced additional interactions, contributing to a more complex bonding network.
- SEM and POM showed a progression from smooth morphologies (P0–P2) to phase-separated, birefringent structures (P3–P5), driven by ChLC’s poor solubility and chiral organization. These optical features are stable up to 120 °C, making P3–P5 promising for stimuli-responsive and colorimetric applications.
- TGA and DSC analyses demonstrated enhanced thermal stability and higher glass transition and melting points in MXene- and ChLC-containing composites. Residual char and increased Tg suggest improved flame retardancy and restricted chain mobility.
- The composites exhibited tunable mechanical behavior. P3 showed optimal stiffness and strength due to MXene-ChLC synergy, while P4–P5 offered greater flexibility attributed to ChLC’s plasticizing effect—favorable for flexible electronics.
- Dielectric analysis revealed P3′s low losses and P4′s high permittivity, driven by structural order and ChLC domains, respectively.

Overall, the PVA-P/MXene/ChLC composite films (P3–P5) demonstrate high efficacy for dielectric, mechanical, and photonic applications, driven by synergistic interactions among components. The triad achieves low dielectric loss (tan δ = 0.109, ε’ = 3.3 in P3) for capacitors, high permittivity (ε’ = 14.3, σ = 1.18 × 10^−8^ S/cm in P4) for EMI shielding, and balanced properties (ε’ = 5.7, Young’s modulus = 1.63 MPa in P5) for flexible sensors. ChLC’s chiral helical structure enhances polarizability and birefringence, while MXene’s conductivity (~7750 S/cm) amplifies charge transport via interfacial polarization (FTIR: ~3400 cm^−1^ O–H, ~1100 cm^−1^ Ti–O shifts). ChLC improves MXene dispersion (SEM: reduced agglomeration in Figure 2d–f), and MXene stabilizes ChLC microdomains, mitigating phase separation. PVA-P’s crosslinked matrix (FTIR: 1087, 844 cm^−1^ P–O–C) ensures structural stability, balancing MXene’s rigidity (P3: 5.88 MPa) and ChLC’s plasticization (P4, P5: 1.63–2.045 MPa). This synergy yields special efficacy, surpassing binary PVA-P/MXene (P1, P2: low ε’ = 5.2–4.8, σ = 10^−10^ S/cm) or PVA-P/ChLC systems (high losses, brittle fracture). The triad validates the hypothesis that combining a phosphorylated polymer, conductive MXene, and chiral ChLC can produce multifunctional composites with tailored properties for capacitors, EMI shielding, and photonic devices, offering a novel approach compared to prior polymer/LC or polymer/MXene systems.

## Figures and Tables

**Table 1:** The composition of the PVA-P film, PVA-P/MXene, and PVA-P/MXene/ChLC composites.

Sample	PVA-P (mL) ^1^	PVA-P (%) ^2^	MXene (mg)/%	ChLC (mg)/%
**P0**	5	100	-	-
**P1**	5	96	10/4	-
**P2**	5	92	20/8	-
**P3**	5	92	10/4	10/4
**P4**	5	82	10/3	50/15
**P5**	5	79	20/6	50/15

^1^ A 5 mL solution PVA-P of concentration 10% *w*/*v*; ^2^ mass percentage relative to the total solid content in the mixtures.

**Table 2:** Thermal data of the composite films (P0–P5).

Sample Code	TGA Data ^1^									DSC Data ^2^	
	*T*_0_	*T*_10%_	*T*_1_	*T*_2_	*T*_3_	*T*_4_	*T_max_*_1_	*T_max_*_2_	*%* Residue	*T_g_*	*T_m_*
**P0**	128	223	113	167	210	–	336	450	15.02	51	–
**P1**	115	242	108	162	201	–	373	448	13.53	74	–
**P2**	129	250	108	166	203	–	365	445	17.77	66	–
**P3**	109	216	112	161	205	286	373	446	12.25	74	–
**P4**	108	223	112	157	206	304	360	444	10.36	70	112
**P5**	108	216	106	157	203	292	364	444	11.71	68	113

^1^ *T*_0_: initial decomposition temperature; *T*_10%_: decomposition temperature corresponding to 10% weight loss; *T*_1–4_: different steps of weight loss rate; *T_max_*_1_: first step of maximum weight loss rate; *T_max_*_2_: second step of maximum weight loss rate; % residue at 650 °C; ^2^ *T_g_*: glass transition temperature; *T_m_*: melting temperature; *T*: temperature values in °C.

**Table 3:** Mechanical properties of the P0-P5 composite films.

Sample	Y ^1^	σ_ult_^2^	ԑ_f_^3^	ԑ_y_^4^	σ_0.2%_^5^	M_R_^6^	M_T_^7^	AE ^8^	PF ^9^
	MPa	MPa	%	%	MPa	kJ/m^3^	kJ/m^3^	kJ/m^3^	N
**P0**	4.85	40.18	49.00	8.27	33.5	168.25	1417.92	1586.17	24.48
**P1**	3.57	29.96	80.04	6.38	21.07	64.55	2021.69	2086.24	40.86
**P2**	3.85	33.47	76.48	8.00	29.41	117.97	2252.19	2370.16	44.59
**P3**	5.88	46.05	42.10	7.60	47.00	201.44	1608.01	1809.45	29.81
**P4**	1.63	16.96	24.11	2.00	5.65	5.85	311.31	317.76	23.31
**P5**	2.05	18.00	21.60	7.18	13.75	48.39	250.20	298.59	29.20

^1^ Y: Young’s modulus (a measure of material stiffness or resistance to elastic deformation); ^2^ σ_ult_: ultimate tensile strength (the maximum stress a material can withstand while being stretched or pulled before it breaks); ^3^ ԑ_f_: fracture strain or ductility (refers to the extent to which a material can stretch or elongate before it breaks); ^4^ ԑ_y_: yield strain (the amount of strain (deformation) a material experiences at the yield point, which is when it begins to deform permanently under stress); ^5^ σ_0.2%_: yield stress (the amount of stress a material can withstand before it begins to permanently deform); ^6^ M_R_: modulus of resilience (the energy a material can absorb elastically without permanent deformation); ^7^ M_T_: modulus of toughness (the total energy absorbed before fracture, indicating overall toughness); ^8^ AE: absorbed energy (absorbed energy until fracture); ^9^ PF: penetration force (the force required to puncture the material).

**Table 4:** The electrical properties of the PVA-P film, PVA-P/MXene, and PVA-P/MXene/ChLC composites.

Sample	ε′ ^a^	ε″ ^b^	tan (δ) ^c^	σ ^d^ [S/cm]
**P0**	11.1	5.55	0.498	5.50 × 10^−9^
**P1**	5.2	1.03	0.197	6.50 × 10^−10^
**P2**	4.8	1.14	0.236	7.23 × 10^−10^
**P3**	3.3	0.35	0.109	2.24 × 10^−10^
**P4**	14.3	18.76	1.307	1.18 × 10^−8^
**P5**	5.7	3.06	0.538	1.93 × 10^−9^

^a^ dielectric permittivity; ^b^ dielectric loss; ^c^ loss tangent, and ^d^ conductivity.

## Data Availability

The original contributions presented in this study are included in the article/Supplementary Materials. Further inquiries can be directed to the corresponding author.

## Supplementary Materials

The following supporting information can be downloaded at: https://www.mdpi.com/article/10.3390/nano15161251/s1.
