# Supplementary material for: Multifaceted Nanocomposites Combining Phosphorylated PVA, MXene, and Cholesteric Liquid Crystal: Design and Application Insights
Source: Nanomaterials (Basel). 2025 Aug 14;15(16):1251. doi: 10.3390/nano15161251 (PMC12388091; doi:10.3390/nano15161251)
Supplement: Supplementary file 1 [file nanomaterials-15-01251-s001.zip › nanomaterials-3756004-supplementary.pdf]

Multifaceted Nanocomposites Combining Phosphorylated PVA, MXene, and Cholesteric Liquid Crystal: Design and Application Insights

Tăchiță Vlad-Bubulac, Diana Serbezeanu, Elena Perju, Dana Mihaela Suflet, Daniela Rusu, Gabriela Lisa, Tudor-Alexandru Filip and Marius-Andrei Olariu

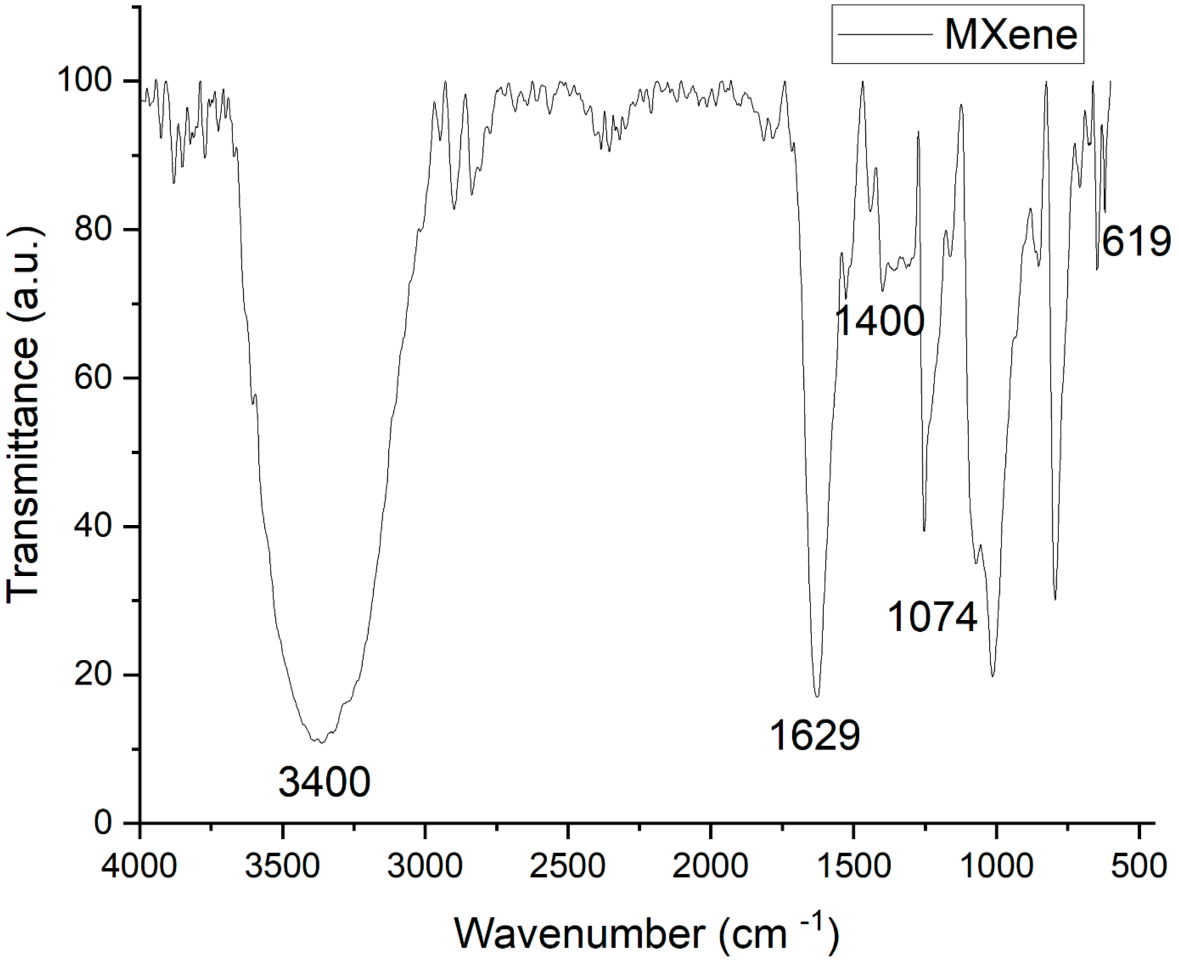

Figure S1. FTIR spectrum of the MXene utilized in the study

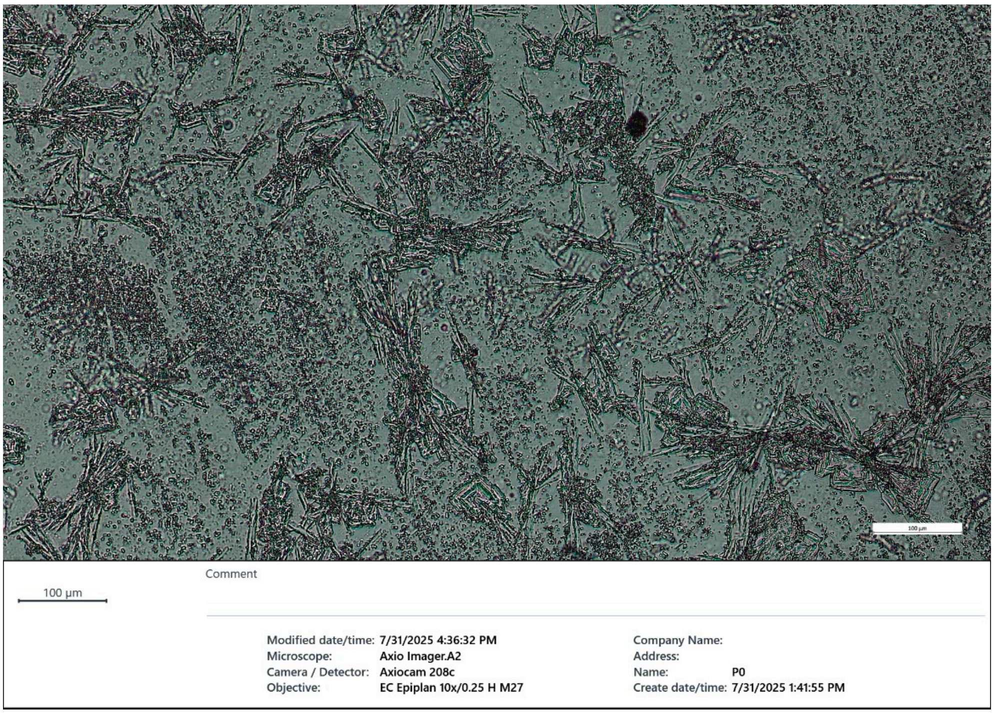

Figure S2. POM image of sample P0

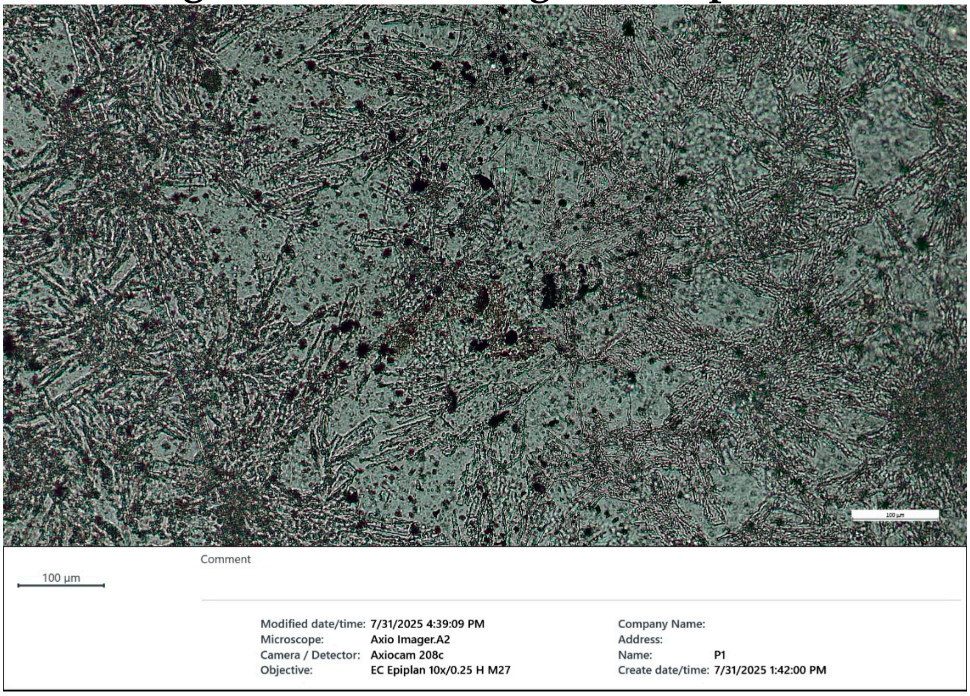

Figure S3. POM image of sample P1

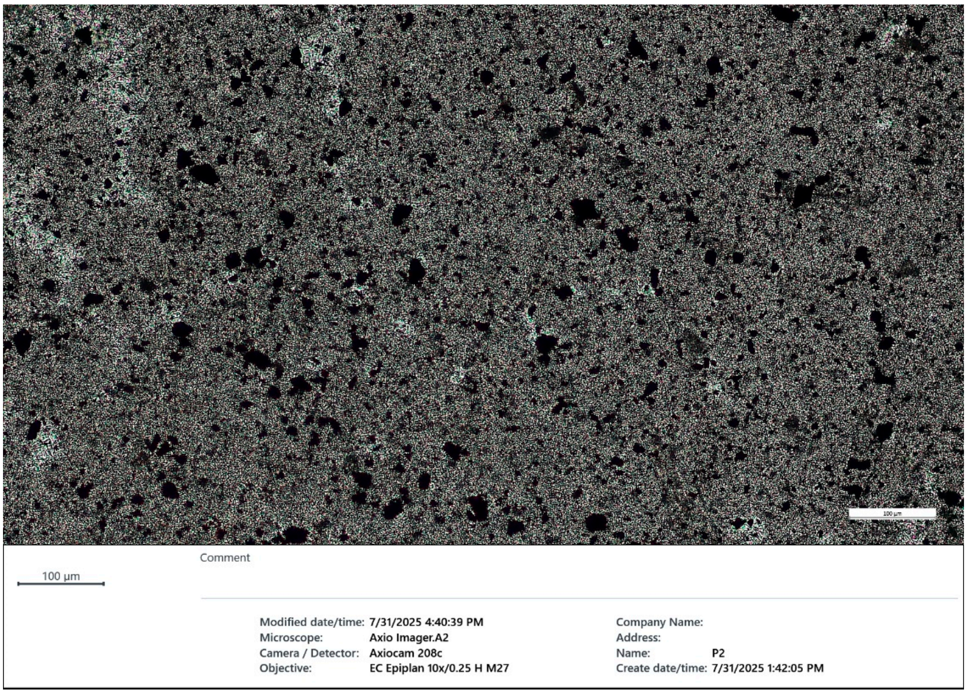

Figure S4. POM image of sample P2

Supplementary Note 1:

The thermal behavior of the PVA-P/MXene/ChLC composites (P3–P5) was compared to their neat components to assess synergistic effects. Pure $Ti_3C_2T_x$ MXene typically begins oxidizing in air around 200–300 °C, forming TiO_2 and CO_2, while lower-temperature mass loss (100–150 °C) is associated with degradation of surface terminations such as –OH, –O, and –F (consistent with FTIR bands at ~3400 and ~1100 cm^{-1}). In inert atmospheres, MXene remains stable up to 500–800 °C; however, its thermal performance may be hindered by aggregation or restacking in the absence of a stabilizing matrix. Cholesteryl acetate (ChLC), a representative cholesteric liquid crystal, undergoes a melting transition at ~110–116 °C (confirmed by DSC in sample P4), followed by an isotropic phase transition between ~150–200 °C. Thermal degradation typically initiates between 250–300 °C in air, primarily due to the decomposition of the cholesterol backbone and acetate group, as reflected by FTIR bands at ~2960–2940 and ~1375 cm^{-1}. Given ChLC's low thermal robustness and phase sensitivity, embedding within a polymer matrix is essential to maintain its structural and functional integrity at elevated temperatures. On the other hand, PVA-P (P0) degrades at ~300–350°C due to its cross-linked phosphate network (FTIR: 1087, 844 cm^{-1} P–O–C). The triad composites exhibit enhanced thermal stability, with an inferred TGA onset of ~350–400°C, surpassing neat components. This is attributed to MXene's thermal barrier effect, delaying PVA-P and ChLC degradation, and ChLC's stabilization within microdomains (POM: birefringent

textures) via interactions with MXene and PVA-P (FTIR: ~3400 cm^{-1} O–H, ~1100 cm^{-1} Ti–O shifts). The composites maintain dielectric (ε' = 3.3–14.3, tan δ = 0.109–1.307), mechanical (Young's modulus: 1.63–5.88 MPa), and optical (birefringence up to 120°C) properties beyond ChLC's Tm, unlike neat ChLC (phase instability) or MXene (oxidation). Reduced low-temperature mass loss, due to matrix encapsulation and interfacial bonding, further enhances durability. These synergistic effects demonstrate that the triad outperforms the sum of its parts, enabling robust performance for capacitors (P3), EMI shielding (P4), and photonic devices (P3–P5).
